# Brain Connectome Graphs based Machine Learning for Diagnosis of Alzheimer’s Disease

**DOI:** 10.1002/alz.090237

**Published:** 2025-01-03

**Authors:** Ke CHEN, Ying Weng, Tom Dening, Akram A. Hosseini, Guokun Zuo

**Affiliations:** ^1^ University of Nottingham, Ningbo, China, Ningbo China; ^2^ University of Nottingham Ningbo China, Ningbo, Zhejiang China; ^3^ University of Nottingham, Nottingham United Kingdom; ^4^ Nottingham University Hospitals NHS Trust, Queens Medical Center, Nottingham United Kingdom; ^5^ The Ningbo Institute of Industrial Technology (CNITECH) of the Chinese Academy of Sciences (CAS), Ningbo China

## Abstract

**Background:**

Alzheimer’s Disease (AD) is a neurodegenerative disorder characterized by progressive cognitive decline and memory loss. Early and accurate diagnosis of AD is crucial for patient information, advance planning, and potentially effective intervention and treatment. The integration of machine learning techniques with brain connectome graphs, encompassing both structural and functional brain connectomes, can enhance the accuracy and efficiency of AD diagnosis.

**Method:**

We propose a framework for AD diagnosis using both structural and functional brain connectome graphs with machine learning techniques. Our framework comprises three stages: image pre‐processing, brain connectome graph construction, and machine learning‐based AD diagnosis. We use PANDA and fMRIPrep for image pre‐processing and brain connectome graph construction. The two types of derived graphs, namely brain structural and functional connectome graphs, are then used as joint inputs for graph neural network (GNN)‐based models for AD prediction, including its early stage, and mild cognitive impairment (MCI).

**Result:**

The experiments are performed on diffusion magnetic resonance imaging (dMRI) and functional MRI (fMRI) obtained from the Alzheimer’s Disease Neuroimaging Initiative (ADNI) datasets, displaying promising results with high performance in identifying AD, (MCI), and cognitively normal (CN) patients. Explainable artificial intelligence algorithms are also applied to the model’s predictions for visualizing decision strategies.

**Conclusion:**

The findings of our research contribute to the burgeoning field of neuroinformatics by offering a novel and effective approach to AD diagnosis. The integration of machine learning with brain connectome graphs has potential to provide early and accurate identification of individuals at risk of AD, and pave the way for timely interventions and personalized treatment strategies. Moreover, this study sheds light on the intricate connectome‐level changes associated with AD and fosters a deeper understanding of the disease’s pathophysiology. Ultimately, this research has established a significant step towards leveraging advanced computational techniques to enhance our ability to diagnose and manage AD.

